# Operative treatment of periprosthetic fractures of the proximal femur with a contralateral, upside-down LISS plate in elderly patients

**DOI:** 10.1186/s12877-023-04277-3

**Published:** 2023-10-06

**Authors:** Marc Schmid, Caroline Gurschler-Pavotbawan, Patrick Fries, Method Kabelitz, Michael Dietrich

**Affiliations:** Clinic for Orthopaedic Surgery, Hand Surgery and Traumatology, City Hospital Zurich, Tièchestrasse 99, 8037 Zurich, Switzerland

**Keywords:** Periprosthetic fracture, Geriatric, Osteosynthesis, Femur, Level III, Therapeutic study

## Abstract

**Background:**

Increasing expectancy of life and levels of activity in the growing geriatric population lead to a rising number of prosthetic implants of the hip and consequently the incidence of periprosthetic fractures of the femur increase. The fracture pattern and the possible instability of the stem are a challenge to the orthopaedic surgeon. Treatment options are complete replacement of the implant or a solitary osteosynthesis. The goal of this study was to analyse the feasibility of the operative intervention using a contralateral reversed anatomic distal femoral LISS® locking plate and the radiological and functional outcome in a geriatric cohort.

**Methods:**

We included all patients older than 75 years of age with a Vancouver type B fracture, which have been treated by osteosynthesis using a LISS® (contralateral reversed) plate in our institution in an interdisciplinary ortho-geriatric setting between 7/2013 and 12/2021. Perioperative morbidities, clinical and radiological outcome during follow-up were retrospectively analysed.

**Results:**

During the observed time period, 83 patients (mean age: 88 years (range: 76–103), male/female: 26/57) were treated. Most fractures were Vancouver type B2 (n = 45, 54%) followed by B1 (n = 20, 24%) and B3 (n = 18, 22%). The most prevalent postoperative surgical complication was anaemia (n = 73, 88%) followed by infections (n = 12, 14%, urinary infections, pneumonia) and cardiovascular decompensation (n = 8, 10%). Clinical and radiological follow up 6–8 weeks postoperative was possible for 59 patients (70%). The majority of them did not describe pain (n = 50, 85%) and had a good or excellent radiological outcome. Three cases needed revision surgery due to infection and another three due to non-union, loosening of the stem or an additional fracture. 1-year mortality was 30%.

**Conclusion:**

We are convinced that the reversed contralateral LISS-plate is an easy-to-use implant with a small complication rate but a very successful and high healing rate in a geriatric, polymorbid cohort.

## Background

Life expectancy is increasing. In 2019 the global life expectancy was 72.8 years. 1990 it was nine years less and it is expected to grow further till 2050 to 77.2 years. In 2022 the share of people with an age of 65 years and older was 10% and is expected to rise to 16% in 2050 [1]. Due to an aging population the number of femoral neck fractures and osteoarthritis of the hip will increase. Over the last ten years the number of annually implanted total and partial hip prosthesis have more than tripled. After revision due to infection, periprosthetic fracture is the second most common reason for revision surgery [2]. After total hip arthroplasty, the ten-year probability for the occurrence a proximal periprosthetic femoral fracture is estimated to be 0.64% and up to 2.25% in high-risk patients [3]. These types of fractures are often accompanied with an increased rate of complications for elderly patients with a high one-year mortality rate and a therefore economic impact [4–6]. The economic impact for a revision hip arthroplasty are 50% higher compared to a femoral osteosynthesis [7].

**Fig. 1:**
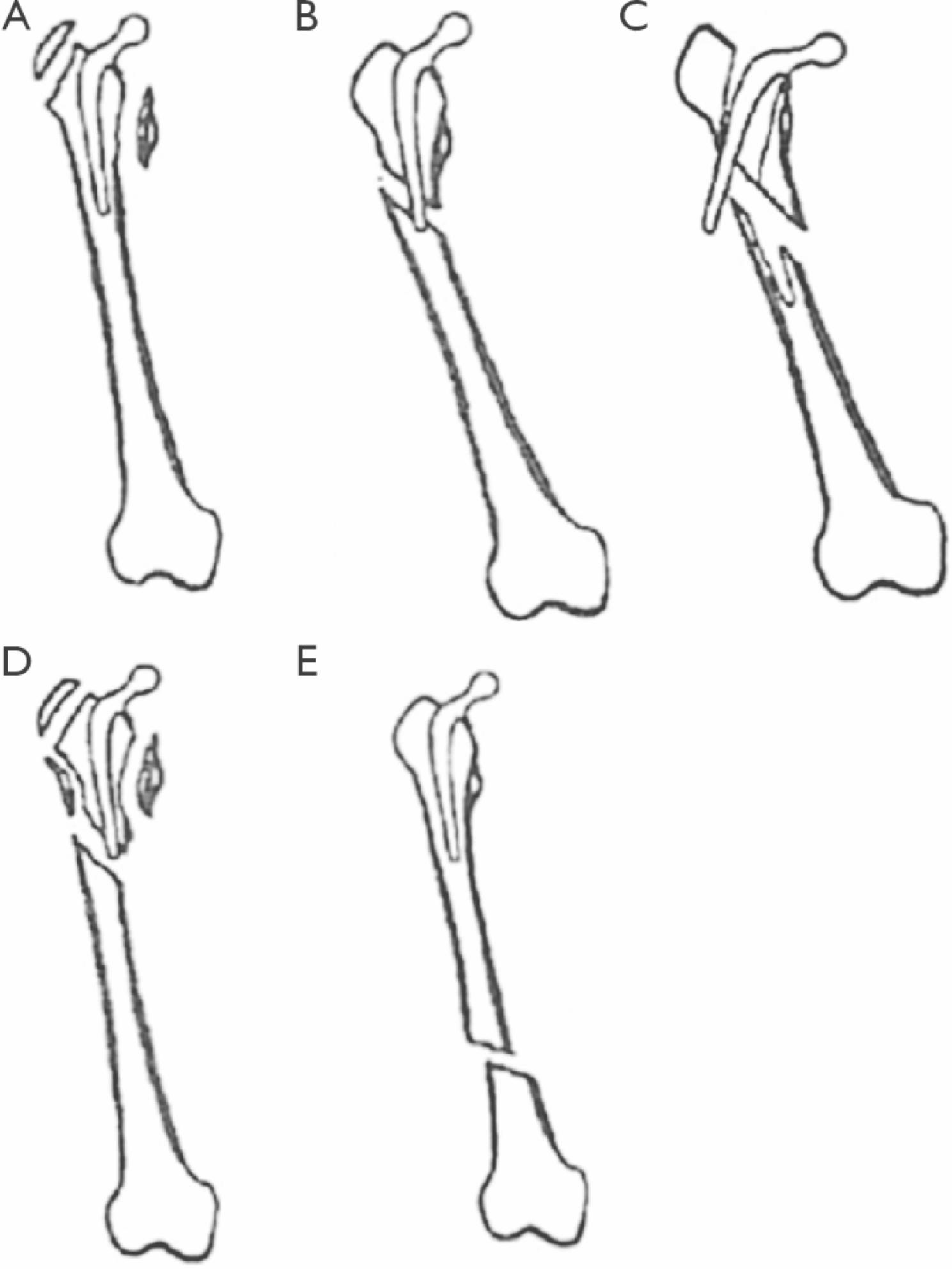
Vancouver classification. (**A**) Vancouver A; (**B**) Vancouver B1; (**C**) Vancouver B2; (**D**) Vancouver B3; (**E**) Vancouver C [8]

Periprosthetic fractures of the hip are commonly categorized according to the Vancouver classification (Fig. 1) [8]. The recommended treatment for displaced type A, B1 and C fractures is an open reduction and internal fixation. Type B2 and B3 fractures can be treated with a replacement of the prosthesis with a longer implant to bridge the osseous defect [9]. Despite the recommended treatment pathways, in some cases, special individual circumstances can lead to different surgical treatment options. As previously described in the literature, in geriatric patients, facing multi-morbidity, an osteosynthesis is an eligible method despite an instable situation of the stem. One possibility to treat non-displaced and displaced Vancouver Type B fractures with or without a loosened stem is the application of a plate osteosynthesis using a reversed anatomic distal femoral Less Invasive Stabilization System (LISS®, DePuy Synthes, Oberdorf, Switzerland) locking plate of the contralateral side. The aim of this retrospective single centre case series was to look at the surgical feasibility of this technique and to analyse the radiological and subjective patient outcome in a geriatric cohort.

## Methods

We retrospectively analysed all patients in the time period of September 2013 until December 2021 which fulfilled the following inclusion criteria.

**Fig. 2:**
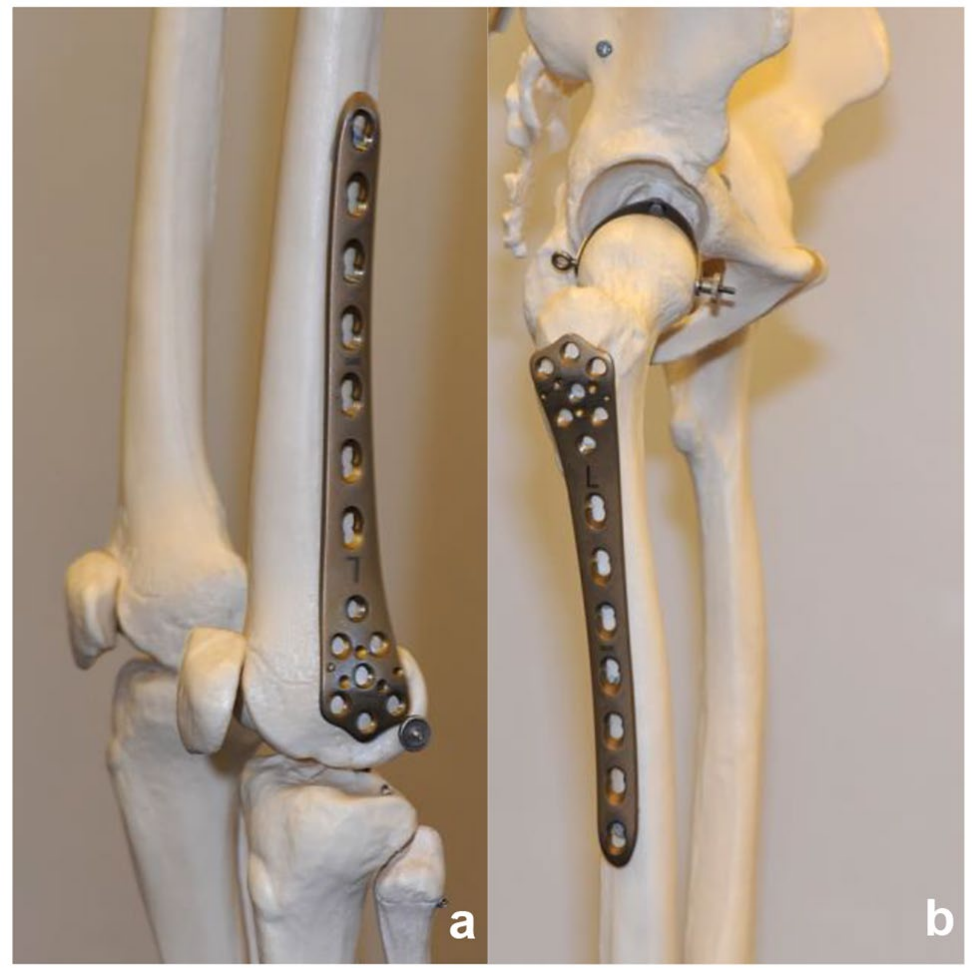
LISS*®* plate on a skeletal model; (**a**) plate for the left distal femur on a left distal femur; (**b**) plate for the left distal femur on a right proximal femur

1. Age over 75 at the time of the proximal femoral periprosthetic fracture,
2. Type B fracture according the Vancouver classification.
3. Fracture treatment with an osteosynthesis using a reversed anatomic distal femoral Less Invasive Stabilization System (LISS®) locking plate with or without additional wire or cable cerclages as shown in Fig. 2. Additionally, we assured that the prosthetic components were not exchanged. During this period of time, we did not perform any revision arthroplasties for fracture treatment.

For the general data and patient characteristics (e.g. age, gender, comorbidities) a retrospective review of the patients electronic records in the local database was performed. To determine the general health status of the patients the Charlson-Index [10] and the ASA (American Society of Anaesthesiologists) score [11, 12] were used. Fractures were classified according to the Vancouver classification. The stability of the prothesis was determined by the well-known radiological signs [13] and later on revised intraoperatively.

### Surgical technique

**Fig. 3:**
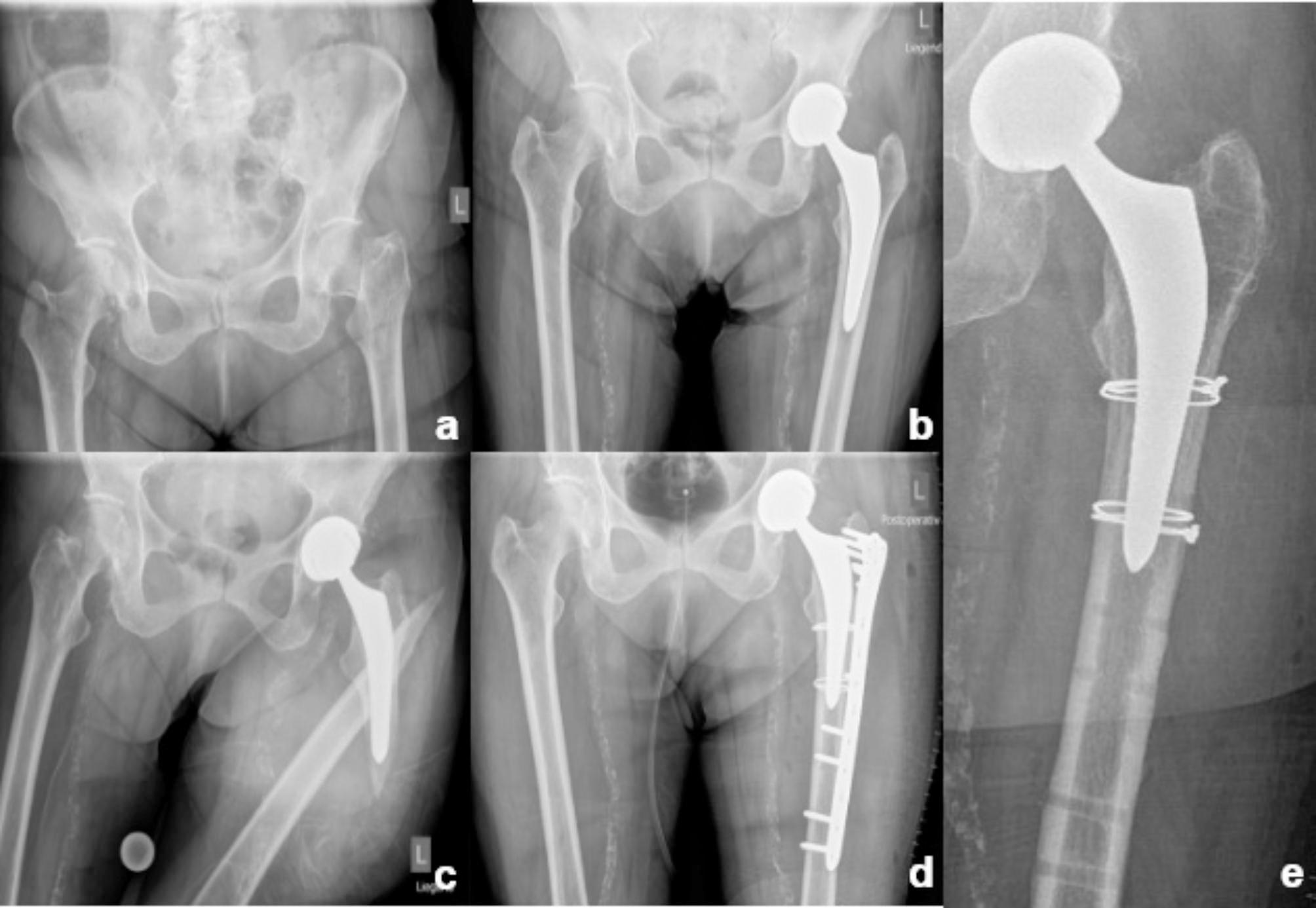
Conventional x-rays; (**a**) displaced femoral neck fracture of the left side. (**b**) treatment with uncemented hip hemiarthroplasty, (**c**) periprosthetic fracture (Vancouver B2), (**d**) osteosynthesis with reversed contralateral LISS plate and wire cerclages, (**e**) after removal of the plate

The patient was positioned in a lateral decubitus on the contralateral side. The subvastus approach to the fractured femur was executed including ligation of the perforating vessels. Depending on the exposure the vastus lateralis muscle was partially detached from the greater trochanter. Care was taken to not fully detach the soft tissue off the fragments. The fracture was subsequently reduced by using reduction forceps, Kirschner-wires or cables for temporary fixation. The reduction of the fragments was controlled under fluoroscopy intraoperatively. After reduction of the fracture, a reversed contralateral angular stable LISS® locking plate was attached using bicortical conventional or locking screws to gain a stable construct. If needed, wire- or cable cerclages were applied to improve stability (Fig. 3). Wound closure was conducted respecting the anatomic layers. The time periods between implantation and fracture, injury and surgical intervention as well as the duration of the operation were documented.

### Postoperative treatment

Depending on the intraoperative stability and quality of the bone, the patients were allowed postoperative full weight bearing (n = 34, 41%) or partial weight bearing (n = 49, 59%) for six to eight weeks with crutches or a walker. The patients received low-molecular-weight heparin for six weeks postoperatively. Plain radiographs were conducted prior to discharge. The duration of the hospitalisation and the post-hospital situation were recorded.

### Follow-up examinations

Routine clinical and radiological follow-up examinations took place six to eight weeks, three-, six and twelve-months postoperatively. Fracture union was evaluated in plain radiographs of the pelvis and a lateral hip view.

Postoperative surgical complications (postoperative blood transfusion, wound infection, revision surgery) as well as non-surgical complications (urinary/pulmonary infections, delirium, cardio-pulmonary decompensation) were documented.

The clinical outcome was evaluated by reviewing the follow-up medical records concerning individual report of pain or functional impairment. To objectify the radiological outcome, we analysed the bony consolidation according the criteria published by Beals and Tower [14]. They graded the outcome into excellent (Arthroplasty: stable; Fracture: healed, minimal deformity, no shortening), good (Arthroplasty: stable, subsidence; Fracture: healed, moderate deformity, moderate shortening) or poor (Arthroplasty: loose; Fracture: non-union, sepsis, new fracture, severe deformity, severe shortening).

### Statistics

R (Version 4.3.0, 2023 The R Foundation for Statistical Computing) was used for data processing with the chi-square test. Significance was assumed at a p ≤ 0.05. Baseline data are, if not stated different, presented in mean, and range.

## Results

### Patients’ characteristics

Between 2013 and 2021, 83 patients (mean age: 88 years (range: 76–103 years), male (n = 26, 31%), female (n = 57, 69%) with a proximal periprosthetic femoral fracture were treated by using a contralateral reversed LISS plate osteosynthesis.

Important patients’ characteristics and results from the follow-ups are shown in Table 1.

Patients had an average of three relevant secondary diagnosis such as malnutrition (n = 59, 71%), cardiovascular disease (n = 55, 66%), dementia (n = 39, 47%), renal disease (n = 32, 39%), gait abnormality (n = 27, 33%), malignoma (n = 12, 14%), pulmonary disease (n = 11, 13%) or diabetes mellitus (n = 10, 12%). The patients were poly-morbid with an average Charlson-Index of 7 ranging from 3 to 19. The ASA score was grade 2 in 18 (22%) patients and grade ≥ 3 in 65 (78%) patients.

Most fractures were type B2 (n = 45, 54%) according to the Vancouver classification followed by type B1 (n = 20, 24%) and type B3 (n = 18, 22%). Twelve (14%) implants of the hip were cemented. One third (n = 27; 33%) of the implants were hemiarthroplasties, whereas 56 (67%) patients had a total hip arthroplasty.

**Fig. 4:**
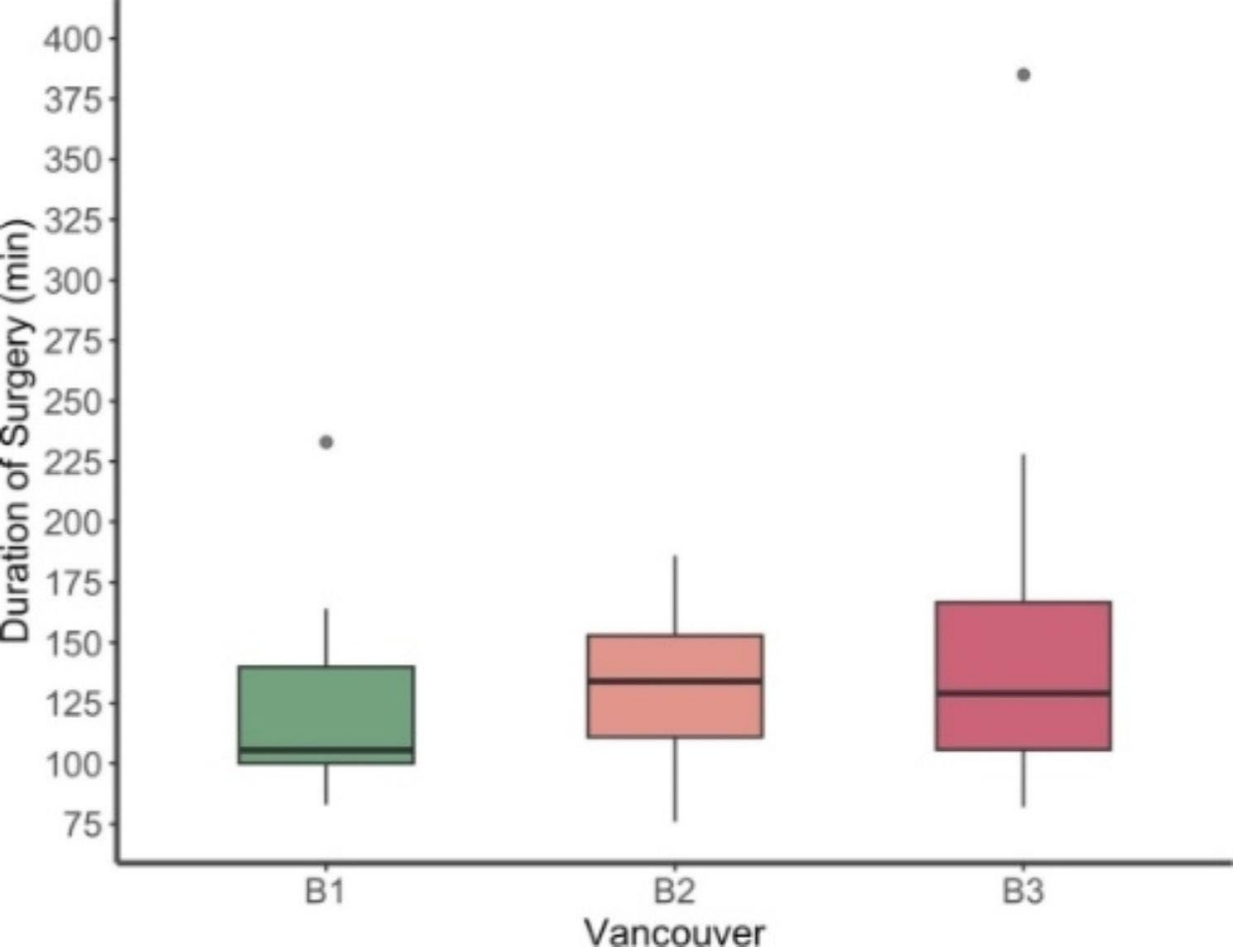
Duration of Surgery in each group of Vancouver B fractures

The average time from primary arthroplasty to the periprosthetic fracture was 9 years (range 0–27). In one quarter of the cases (n = 21, 25%), the fracture occurred in the first two years. In 10% (n = 8) the fracture even occurred in the first three weeks postoperatively. In seven out of the eight early fractures (88%), the prosthesis was uncemented. There were significant more early onset fractures in uncemented compared to cemented arthroplasties (p = 0.001). Thirty (36%) fractures were diagnosed at least ten years after implantation of the arthroplasty. The operation was hold within the first 24 h after the injury in 30 patients (36%), within 24 to 48 h in another 30 patients (36%) and 23 patients (28%) were operated after more than 48 h. The average operating time was 134 min (range 76–228 min). Vancouver type B3 fractures showed no statistical difference in duration of surgery compared to Vancouver B2 (p = 0.950) or B1 (p = 0.600) fractures (Fig. 4). The mean duration of hospital stay was 10.5 days (range 4–34 days), after which most patients (n = 46, 55%) were discharged into a nursing home followed by geriatric unit (n = 22, 27%), rehabilitation (n = 8, 10%) and home (n = 2, 2%). Five patients (6%) died during the hospital stay.

### Surgery-related complications and revision surgery during the hospitalisation

**Fig. 5:**
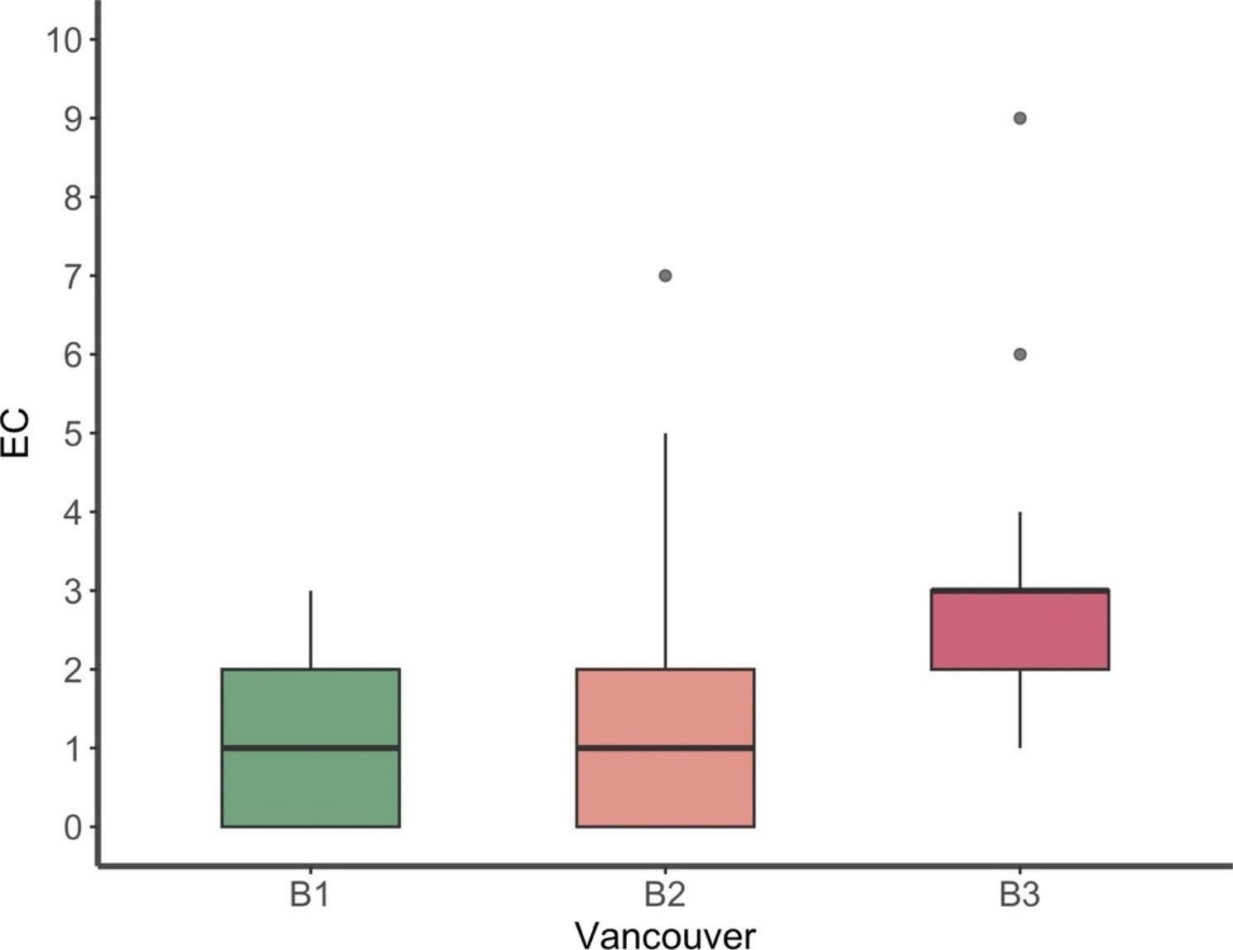
Amount of packed red blood cells (EC = Erythrocyte concentrate) given in Vancouver B1, B2 and B3 fractures

The most common documented postoperative surgical complication was anaemia (n = 73, 88%), which had to be substituted in 61 patients (73%) with an average substitution of 1.6 (range: 0–9) unit of packed red blood cells. The preoperative haemoglobin was 111 g/l (range: 68–149) compared to 84 g/l (range: 55–120) on the first postoperative day. Vancouver B3 fractures showed a significantly higher need for units of packed red blood cells compared to Vancouver B2 (p < 0.001) and Vancouver B1 fractures (p < 0.001) (Fig. 5).

Three patients developed an early implant-associated infection. In one case, the exchange of the material of the osteosynthesis twelve days after implantation was performed with additional debridement four days later. Another patient had two changes of the plate and cerclages after 19 and 27 days. In the third case, the patient had to undergo five revision surgeries including a hip resection arthroplasty (“girdlestone operation”) followed by reimplantation of a revision stem. The average age of the patients with a postoperative surgical side infection was 86 years (range: 79–92 years) and there was one in each Vancouver type B fracture group.

During the hospital stay, 13 non-surgical infections (16%) were documented, whereas eight (10%) were infections of the urinary tract and five (6%) of the lung. Eight patients showed a status of cardiovascular decompensations (10%). 17 (20%) patients suffered from a postoperative delirium.

**Table 1:** Patient Characteristics subdivided into Vancouver classification

	B1	B2	B3
Number	20	45	18
Sex			
Female	80%	58%	83%
Male	20%	42%	17%
Age ( years)	88	88	88
Time between Prothesis and Fracture (in years)	11	9	8
Hospital stay ( days)	10	10	11
Implant type			
Total Hip Arthroplasty	75%	71%	50%
Hemi-Prosthesis	25%	29%	50%
Cemented			
Yes	25%	11%	11%
No	75%	88%	88%
ASA			
2	15%	31%	6%
3	85%	69%	94%
Charlson Comorbidity Index (mean)	6.6	7.3	6.5
Operation Time ( minutes)	119	133	133
Anaemia	85%	84%	100%
Packed red blood cells ( units)	1.1	1.5	3.1
Revision (infection)	5%	2%	6%
Revision (re-fracture, delayed-union, loosening)	0%	7%	0%
Subsidence	0%	14%	6%
Beals and Tower			
Excellent	100%	73%	85%
Good	0%	15%	15%
Poor	0%	12%	0%
Pain at follow-up	15%	12%	23%
1-year Mortality	35%	33%	17%

### Follow-up examinations and revision surgery

Until May 2022, 45 of the included 83 patients were already deceased, with a 3-month mortality rate of 22%, a 1-year mortality rate of 30% and a 3-year mortality rate of 42%. Five of those died during the hospital stay. The mortality rate was not significantly higher in Vancouver B2 (OR 0.4; 95%-CI [0.1–1.4]; p = 0.182) or Vancouver B3 (OR 0.9; 95%-CI [0.1–5.4]; p = 0.888) compared to Vancouver B1.

The first clinical and radiological follow-up after an average of 53 days postoperative (range 34–108 days) was possible in 59 cases (70%). Twenty-four patients were lost to follow-up due to various reasons. Seventeen individuals (20%) died during the time until first follow-up consultation after a mean time of 31 days. Two (2%) were not able to attend the outpatient clinic for follow-up examinations due to reduced general conditions. One patient (1%) was treated with a resection arthroplasty due to an infection. One patient (1%) presented with an additional periprosthetic fracture distal to the osteosynthesis and was therefore not able to attend to follow-up examinations. Three cases (4%) were lost to follow-up due to unknow reasons.

The vast majority of the patients (n = 50, 85%) reported neither to have any functional impairment nor symptoms of pain at time of the latest follow-up. There was no difference in subjectively experienced pain in total hip arthroplasty (OR 0.8; 95%-CI [0.2–4.1]; p = 0.745) compared to hemiarthroplasty.

Plain antero-posterior x-rays of the pelvis showed a stem subsidence in seven cases (8%, mean 11 mm, range 4–27 mm). Six of them were in Vancouver type B2 fractures and one in Vancouver type B3 fractures. Vancouver type B2 fractures had significantly higher probability for subsidence compared to Vancouver B1 (p = 0.001) and Vancouver B3 (p = 0.029). According to Beals and Tower, there were 81% (n = 49) excellent, 12% (n = 7) good and 7% (n = 4) poor radiological results. Poor radiological outcomes showed no difference in duration of surgery compared to excellent (p = 0.200) and good outcomes (p = 0.63). There was no difference in individually experienced pain in good outcomes (OR 1.2; 95%-CI [0.1–8.9]; p = 0.879) compared to excellent outcomes. However, there was a significantly higher risk for subjectively experienced pain in poor outcomes (OR 14.3; 95%-CI [1.2-338.3]; p = 0.0405) compared to excellent outcomes. One of the four individuals with a poor radiological outcome had a stem subsidence of 27 mm but did not want a revision due to the advanced age of 96 years. The other three cases with poor outcomes are described below.

Revision surgery was necessary in three further cases. Once because of substantial loosening of the stem with a complete fracture consolidation after 221 days. The material of the osteosynthesis was removed and the shaft of the prothesis was changed. Postoperatively, another re-fracture occurred leading to further need for osteosynthesis four days later. One patient showed a delayed-union with an atraumatic dislocation of the plate and proximal screws after 71 days. A re-osteosynthesis with additional cerclages and new proximal screws was needed. In the third case, the.

reintervention was needed due to an additional trauma leading to a new periprosthetic fracture distal to the first osteosynthesis 45 days after the first surgery. The average age of the patients with revision surgery was 85 years (range: 82–89 years) and all of them presented with a Vancouver type B2 fracture.

In one case, plate removal was performed on demand without any signs for implant failure or infection (Fig. 3.).

## Discussion

To our knowledge, the present study is the first to describe the clinical and radiological outcome after osteosynthesis of proximal femoral periprosthetic fracture using a contralateral reversed LISS plate in a geriatric cohort. We were able to show that the operative treatment of Vancouver type B1-3 fractures using this construct is a viable option and leads to good clinical as well as the radiographic results.

This study has limitations. First and most important is the lack of a control group and standardized clinical evaluation at the follow-up examination. Additionally, only 70% of the patients could return for follow-up examinations due to a high mortality in an old polymorbid cohort. This leads to a decreased informative value concerning the presented data. Furthermore, the retrospective study design has its well-known disadvantages.

Due to the very high age and comorbidities of the patients, we saw the expected high rate in mortality and morbidity despite the fact that all patients were treated interdisciplinary in an ortho-geriatric ward. According to the Trustees Report 2022 of the Social Security Administration of the USA the normal 1-year mortality at an age of 86 years is approximately 10%. Depending on the treatment option and the age the 1-year mortality after a hip fracture varies in the literature between 7.8 and 95% [15, 16]. The mortality in our patients was three times higher, which can be explained due to the high comorbidities. Considering the average Charlson Comorbidity Index [10] of seven the mortality is equivalent to the literature, which is 28% for a Charlson Comorbidity Index of ≥5 [17] There was a significantly higher risk for mortality with increasing age. Several studies [18–20] showed no significant difference in the 1-year mortality between open reduction and internal fixation and revision arthroplasty. Furthermore, we could show that there is no significant increase in mortality in Vancouver B3-fracture compared to Vancouver B1- and B2-fractures. Additionally, the results show, that the subjectively experienced pain did not differ between B1- and B2 fractures but showed an increase in B3 fractures.

The distribution of fracture types was approximately according to the literature with 24% B1-, 54% B2- and 22% B3-fractures [4].

Because there were significantly more early onset fractures in uncemented arthroplasties than in the cemented ones, one could assume, the fracture may already occurred during the operation.

By using only an osteosynthesis without a revision of the arthroplasty there is a risk of a loosened stem and of a subsidence of the stem. 8% of our patients had a subsidence and all of them occurred in Vancouver type B2 and B3 fractures. Beside this fact, the described type of osteosynthesis led to a high rate of good radiological outcomes in our follow-up group with a much higher number of excellent result (81%) according to Beals and Tower [14] compared to other studies [20, 21].

However, there were three revisions needed, one due to a painful loosened stem, one due to a re-fracture and one due to a delayed union. All of them were in Vancouver B2 fractures. The revision rate due to a failed revision method in Vancouver type B2 and B3 fractures was 5%. An additional 3% needed revision due to a surgical side infection. A systematic review compared several case series of different treatments of Vancouver B2 and B3 fractures. They showed a revision rate of 13.3–28.6% in the groups of osteosynthesis alone without revision of the stem and a revision rate of 12.4–14.4% in the groups which included revision of the stem [22]. Compared to the treatment with other implants, our data revealed an almost 2 to 4 times reduction of the revision rate with a revision rate of 8% in Vancouver type B2 und B3 fractures.

Additionally, the average operation time in our cases was 129 min. Meanwhile the average operation time according to the literature for revision arthroplasty for proximal periprosthetic femur fracture is 160–182 min [23, 24]. The radiological outcome was not related to the surgery time. Also, there was no significant difference in surgery time in the different fracture types. Moreover, the need of packed red blood cells perioperatively was (1.6 units) significantly lower compared to revision arthroplasty in the literature (5.1 units) [19]. In our cohort, Vancouver B3-fractures showed a significant higher number of packed red blood cells needed compared to B1- and B2-fractures.

Compared to many other studies, even though our cohort is older and has more comorbidities, the mortality and complication rate is comparable.

## Conclusion

In this retrospective study we have shown that the treatment of a periprosthetic proximal femur fracture using a reversed contralateral less invasive plate system leads to good to excellent clinical and radiological outcome in a geriatric cohort. Especially in times where storage room is limited and different preshaped implants for every region is costly, this implant can be used in the described way for distal femur fractures as very effectively for periprosthetic proximal femur fractures.

Nevertheless, the expected increased rate of complications and mortality have been confirmed for this difficult and polymorbid cohort. For this vulnerable group of patients different treatment options have to be taken into account considering, that, next to revision arthroplasty, osteosynthesis is always a feasible option. Further prospective or comparative studies are needed to confirm the described outcomes.

## Data Availability

The datasets used and/or analysed during the current study are available from the corresponding author on reasonable request.
